# The Diversity and Dynamics of Sex Determination in Dioecious Plants

**DOI:** 10.3389/fpls.2020.580488

**Published:** 2021-01-15

**Authors:** Ana Paula Leite Montalvão, Birgit Kersten, Matthias Fladung, Niels Andreas Müller

**Affiliations:** Thünen Institute of Forest Genetics, Großhansdorf, Germany

**Keywords:** dioecy, monoecy, hermaphroditism, sex determination via one gene, sex determination via two genes, sex chromosomes, sex-determining region

## Abstract

The diversity of inflorescences among flowering plants is captivating. Such charm is not only due to the variety of sizes, shapes, colors, and flowers displayed, but also to the range of reproductive systems. For instance, hermaphrodites occur abundantly throughout the plant kingdom with both stamens and carpels within the same flower. Nevertheless, 10% of flowering plants have separate unisexual flowers, either in different locations of the same individual (monoecy) or on different individuals (dioecy). Despite their rarity, dioecious plants provide an excellent opportunity to investigate the mechanisms involved in sex expression and the evolution of sex-determining regions (SDRs) and sex chromosomes. The SDRs and the evolution of dioecy have been studied in many species ranging from Ginkgo to important fruit crops. Some of these studies, for example in asparagus or kiwifruit, identified two sex-determining genes within the non-recombining SDR and may thus be consistent with the classical model for the evolution of dioecy from hermaphroditism via gynodioecy, that predicts two successive mutations, the first one affecting male and the second one female function, becoming linked in a region of suppressed recombination. On the other hand, aided by genome sequencing and gene editing, single factor sex determination has emerged in other species, such as persimmon or poplar. Despite the diversity of sex-determining mechanisms, a tentative comparative analysis of the known sex-determining genes and candidates in different species suggests that similar genes and pathways may be employed repeatedly for the evolution of dioecy. The cytokinin signaling pathway appears important for sex determination in several species regardless of the underlying genetic system. Additionally, tapetum-related genes often seem to act as male-promoting factors when sex is determined via two genes. We present a unified model that synthesizes the genetic networks of sex determination in monoecious and dioecious plants and will support the generation of hypothesis regarding candidate sex determinants in future studies.

## Introduction

Contrary to most animals, hermaphroditism occurs widely in plants (Westergaard, 1958; Bawa, 1980; Renner, 2014). However, the separation of the sexes, either in different locations of the same individual (monoecy), or in different individuals (dioecy) has been recognized in varying frequencies across the numerous plant species (Renner and Ricklefs, 1995; Renner, 2014). Unisexual gametophytes are widespread within the bryophyte lineages, with 68% of mosses, 57% of liverwort, and 40% of hornwort species (Villarreal and Renner, 2013). Among the seed plants there is a striking discrepancy: while in gymnosperms 65% of the species are dioecious (Walas et al., 2018), in angiosperms, dioecy is a comparatively uncommon phenomenon, comprising only 5–6% of all species (Renner, 2014). However, even though dioecy is considered rare among flowering plants, its occurrence has been reported in several phylogenetic taxa (around 15,600 species spread over 175 families and 987 genera), suggesting that its evolution occurred independently hundreds if not thousands of times (Westergaard, 1958; Renner, 2014).

**FIGURE 1:**
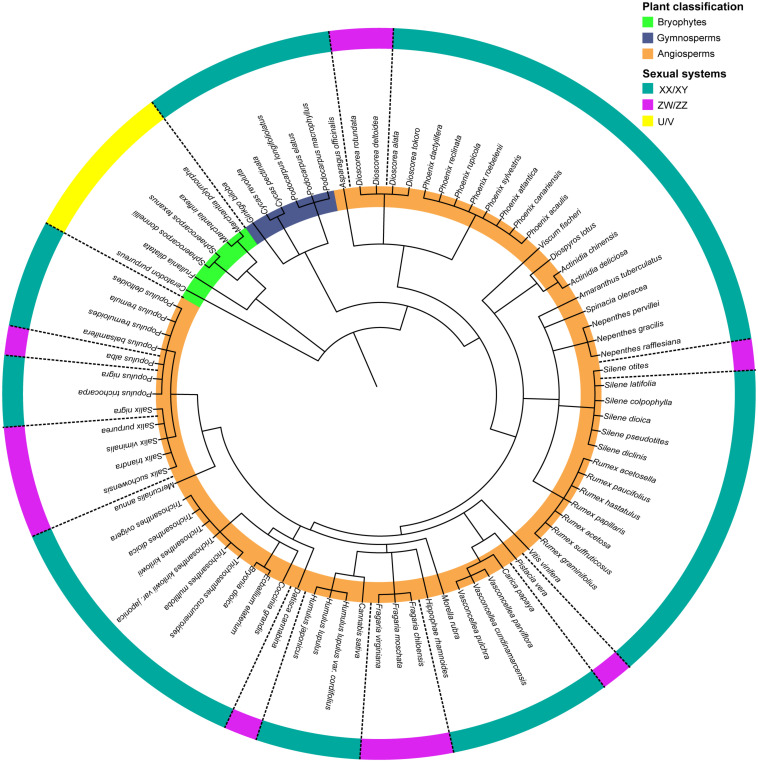
Phylogenetic tree of species with known sex determination systems and sex chromosomes. Plant classification (inner circle): bryophytes (green), gymnosperms (blue), and angiosperms (orange); Sexual systems (outer circle): male heterogametic system XY/XX (green), female heterogametic system ZW/ZZ (pink), and haploid U/V system (yellow). The phylogenetic tree was built using phyloT v2: a tree generator (based on NCBI taxonomy; https://phylot.biobyte.de/). The list of the species with their respective references is presented in the Supplementary Table 1.

In humans, mammals, some insects and several plants, the males are heterogametic, meaning that males carry two different sex chromosomes denoted as X and Y, while females carry two X chromosomes. In many insects, the X:O system exists, where females carry two X chromosomes but the males only one. The second sex chromosome is absent in males. In birds, some reptiles and a few plants, the females are the heterogametic sex, represented by ZW, while males are ZZ. In bryophytes, with a predominant haploid phase, the male sex chromosome is referred to as U while the female is referred to as V (Bachtrog et al., 2014; Capel, 2017; Renner et al., 2017). In contrast to animals, sex chromosomes in plants have been identified in very few species to date (Ming et al., 2011; Renner, 2014; Muyle et al., 2017), especially because of the low number of species with heteromorphic sex chromosomes. Figure 1 presents dioecious plant species with cytogenetic and/or molecular evidence for the presence of sex chromosomes and their sex determination systems in a phylogenetic perspective. Among these plant species, male heterogamety (XY) is predominant (84.7%), while female heterogamety (ZW) only comprises 15.3%.

Interestingly, turnovers in the heterogametic systems have been reported in several plant families and genera. For instance, the genera *Dioscorea, Populus, Salix*, and *Silene* all comprise species with XY and ZW systems (Figure 1). One theory suggested that transitions between heterogametic systems may be selected when the sex chromosome is degenerated and the heterozygous sex has low fitness (Blaser et al., 2014), and another possibility is when sexually antagonistic polymorphism is maintained on an autosome, a new sex-determinant that arises in the region becomes advantageous (Doorn and Kirkpatrick, 2007). Recent findings indicated that these transitions among heterogametic systems can be favored by haploid selection (Scott et al., 2018). Shifts between different sex chromosome systems (XY ↔ ZW) are also well documented across several clades of animal species and appear to be a common phenomenon, especially in reptiles, frogs, and fishes (Vicoso et al., 2013; Bachtrog et al., 2014; Zhou et al., 2014; Jeffries et al., 2018; Pennell et al., 2018; Kottler et al., 2020).

This diversity of sexual systems in plants has an important impact on evolutionary biology as well as importance for seed and fruit production (Renner, 2016). Despite the extensive amount of theoretical work regarding the evolution of dioecy and the possible resulting sex-determining systems, experimental data are only recently becoming available. This review aims to explore and synthesize the diversity of sex-determining mechanisms in several dioecious plant species. A unified model of sex determination is presented and possibly enriched functional categories of sex-determining genes are highlighted.

## Genetic Mechanisms of Sex Determination

The numerous independent evolutionary origins of dioecy suggest that many different genetic and molecular mechanisms are involved in the separation of the sexes in plants (Diggle et al., 2011). Studies in diverse species aimed at clarifying these mechanisms and ultimately explaining the evolution of dioecy and switches between sexual systems. The recent advances in molecular techniques are making this increasingly possible. By identifying and functionally characterizing the genes underlying sex determination, possible evolutionary pathways to dioecy can be inferred.

For dioecy to evolve, either from a hermaphroditic or a monoecious state, at least two changes, i.e., mutations, are necessary. The most influential work proposing a model for the evolution of dioecy, (Charlesworth and Charlesworth, 1978), concludes that the most likely evolutionary pathway from hermaphroditism to dioecy involves two successive mutations with a gynodioecious intermediate. First, a recessive male-sterility mutation gives rise to a gynodioecious population (co-existence of females and hermaphrodites). Second, a dominant female-sterility mutation, which needs to be linked with the first mutation into a region of suppressed recombination, results in dioecy. While it is stated that both mutations might occur in a single gene, the most likely outcome of the “gynodioecy pathway” is two sex-determining genes in the SDR, one regulating female floral organ development and the other one male floral organ development. On the other hand, a single-gene sex-determining system can evolve if the factors regulating female and male function are connected by an epistatic genetic interaction rather than physical linkage (Golenberg and West, 2013).

We will first review studies that indicate two sex-determining genes at the SDR, followed by work indicating a single sex-determining gene.

### Sex Determination via Two Genes

Strong experimental support for two sex-determining genes at the SDR has been shown for kiwifruit (*Actinidia deliciosa)* and asparagus (*Asparagus officinalis)*. Additionally, in date palm (*Phoenix dactylifera)* and grapevine (*Vitis vinifera)* sex determination via two genes appears likely and may thus also be consistent with the “gynodioecy pathway.”

Kiwifruit (*A. deliciosa*) is a major fruit crop with an XY system of sex determination. Studies in kiwifruit demonstrated that two genes are responsible for sex determination, one affecting ovule production, and another one the production of pollen. A male-specific type-C cytokinin response regulator called “*SHY GIRL*” (*SyGI*) was identified as a suppressor of feminization (*Su*^*F*^). The model systems *Arabidopsis thaliana* and *Nicotiana tabacum* were used to validate the functionality of this gene. Transgenic expression of *SyGI* resulted in a stable suppression of carpel development (Akagi et al., 2018). Subsequently, the male-promoting factor (*M*_1_), called “*FRIENDLY BOY*” (*FrBy*) was identified as the second Y-encoded gene responsible for sex determination in kiwifruit, specifically for the development of androecia. This gene is related to the *MICROSPORE AND TAPETUM REGULATOR 1* (*MTR1*) protein family, which, in rice, contributes to tapetum degradation affecting male fertility (Tan et al., 2012). The function of this second gene was validated in model plants as well as in kiwifruit. The artificial introduction of the *FrBy* gene into a female kiwifruit cultivar resulted in hermaphrodites (Akagi et al., 2019).

Similar to kiwifruit, in garden asparagus (*A. officinalis*) two genes were identified as the sex-determining genes: one of which is the Y-specific *SUPPRESSOR OF FEMALE FUNCTION* (*SOFF*) gene, acting as suppressor of femaleness. Experimental validation was achieved using a gamma irradiation knockout that resulted in the conversion of males to hermaphrodites (Harkess et al., 2017). The *DEFECTIVE IN TAPETUM DEVELOPMENT AND FUNCTION 1* (*TDF1*), encoding a MYB transcription factor and expressed only in males, was recognized by different research groups as a strong candidate for sex determination acting as a promoter of male function (Harkess et al., 2017; Murase et al., 2017; Tsugama et al., 2017). Ethyl methanesulfonate (EMS) mutagenesis of *aspTDF1* resulted in the conversion of males to asexual neuters. The knockout of both genes (*SOFF* and *aspTDF1*) converted males to females (Harkess et al., 2020).

These results show functional evidence that two sexually antagonistic genes at the SDR are necessary to determine sex in asparagus and in kiwifruit. Both species reveal distinct male-promoting factors (*FrBy* in kiwifruit and *aspTDF1* in asparagus), yet both having functions in the tapetum which is essential for male flower fertility.

The date palm (*P. dactylifera*), an important commercial fruit crop, presents a male heterogametic system (XY), and all 14 known species from the genus *Phoenix* are dioecious (Cherif et al., 2016). Recent work has identified sex-linked markers and a sex-linked region of ∼6 Mb (Hazzouri et al., 2019) although candidates for sex-determining genes remained unidentified until recently. Torres et al. (2018) uncovered male-specific sequences in 13 species of *Phoenix* whereas no unique female-specific sequences were observed. Candidate genes potentially involved in sex determination in *P. dactylifera* were revealed with similarity to *CYTOCHROME P450* (*CYP450*), ortholog of *CYP703A3* from rice (*Oryza sativa)*, *GLYCEROL-3-PHOSPHATE ACYLTRANSFERASE 3-LIKE* (*GPAT3-like*), an ortholog of *GPAT3* from *A. thaliana* and the gene *LONELY GUY* (*LOG*). The identified genes have known functions in sexual development in other monocot species. Both *CYP* and *GPAT3-like* are expressed only in *Phoenix* males and seem to be important for male flower development and fertility. In rice, both *CYP703* and *GPAT3* are expressed in tapetal cells and have functions in pollen formation and anther development. The deletion of the homologs of *CYP703* in rice, maize, and *A. thaliana* (Morant et al., 2007; Yang et al., 2014) and of *GPAT3* in rice (Men et al., 2017) led to male sterility. *LOG* genes are a family of genes with an important role in cytokinin activation and a potential role for female flower development (Kurakawa et al., 2007). In rice, *LOG* mutants presented flowers without ovules (Yamaki et al., 2011). While the functionality of these genes in date palm remains to be tested, the data are consistent with sex determination via two genes.

All grapevines (*V. vinifera*) are dioecious, however, during domestication, humans have generated a hermaphroditic grapevine subspecies (*Vitis vinifera* ssp. *vinifera*) (Fechter et al., 2012; Coito et al., 2018). Different models have been proposed to explain the genetic basis of sex determination in grapevines, but only recently evidence was put together to help clarifying these hypotheses. A genetic map demonstrated the sex-determining region contains several genes with potential involvement in flower development (Fechter et al., 2012; Picq et al., 2014). Haplotype-resolved genomes of hermaphrodite, female and male grapevines finally resolved the sex-determining region which spans approximately 260 kb on chromosome 2 (Zhou et al., 2019; Massonnet et al., 2020). The gene content and variability were characterized, and candidate genes proposed. Of ten genes with female-specific single nucleotide polymorphisms (SNPs), the *INAPERTURATE POLLEN 1* (*INP1*) gene was revealed as a likely candidate for the male-promoting factor (Massonnet et al., 2020). In *A. thaliana*, *INP1* is necessary for fertile pollen (Dobritsa and Coerper, 2012). The results also showed that all individuals with female flowers were homozygous for an eight bp deletion in *VviINP1* indicating that this may be the causal polymorphism leading to male-sterility. In contrast, all individuals with male flowers carried one functional and one non-functional copy of *VviINP1*. Convincing candidate genes for the dominant female suppressor include the *ADENINEPHOSPHORIBOSYL TRANSFERASE* (*APRT3)*, a cytokinin regulator (Coito et al., 2018; Badouin et al., 2020) and the transcription factor *YABBY3* (Massonnet et al., 2020) that belongs to a gene family previously implicated in the development of carpels in *A. thaliana* (Villanueva et al., 1999). While future studies are necessary to understand the specific roles and connections of these different factors, the current data provide strong evidence for sex determination via (at least) two genes.

### Artificial Generation of Dioecy From Monoecy

Dioecy was artificially engineered in the monoecious species maize (*Zea mays*) and melon (*Cucumis melo*). Nearly a century ago, two genetically interacting genes were identified to control sex expression in monoecious maize: the *TASSEL SEED* (*Ts*) gene, which is a female suppressor, and the *SILKLESS* (*Sk*) gene, which protects female floral organ development from the action of *Ts*. In a *sk* mutant background, a single segregating *ts* mutation could be employed for the artificial production of dioecious maize (Jones, 1934). In the monoecious melon, a network of three genes controls sex expression (Boualem et al., 2008, 2015). *CmACS11* controls the development of pistillate flowers, just like *Sk* in maize. *CmWIP1* suppresses female flower development, just as *Ts* in maize. Finally, *CmACS7* represses male flower development. The *1-AMINOCYCLOPROPANE-1-CARBOXYLIC ACID SYNTHASE* (*ACS*) genes are key enzymes in ethylene synthesis in plants, while *WIP* is a zinc finger transcription factor (Appelhagen et al., 2010). Just as in maize, the artificial conversion from monoecy to dioecy was achieved by producing a population with a non-functional *acs11* gene and a segregating *wip1* mutation (Boualem et al., 2015). Both examples demonstrate a potential pathway for the evolution of dioecy that results in the fixation of one mutation and a single segregating mutation controlling sex determination (Muller, 1932; Westergaard, 1958; Golenberg and West, 2013; Renner, 2016; Cronk and Müller, 2020).

### Sex Determination via One Gene

In several non-plant taxa, a single regulator gene is the primary mechanism for sex determination. For instance, the *SEX-DETERMINING REGION Y* (*SRY*) is the master switch of sex determination in most mammals (Gubbay et al., 1990; Kashimada and Koopman, 2010; Li et al., 2014). Additionally, one single gene, that is *DOUBLESEX AND MAB-3 RELATED TRANSCRIPTION FACTORS* (*DMRT*), can control sex in several different groups of animals (e.g., birds, fish, and frogs) (Hodgkin, 2002; Smith et al., 2009).

**FIGURE 2:**
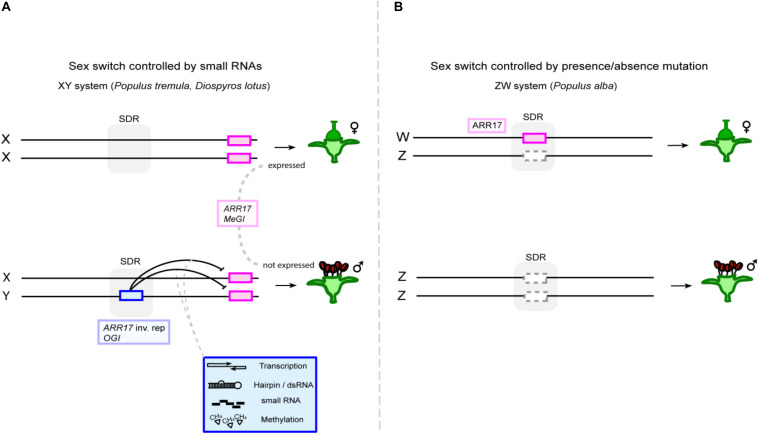
Single-gene sex determination enables turnovers between XY and ZW systems. **(A)** The feminizing sex switch (magenta box), i.e., *ARR17* and *MeGI* in poplar and persimmon, respectively, is located outside of the sex-determining region (SDR, indicated by gray shading), because a hairpin RNA-encoding Y-chromosomal sequence (blue box) controls its activity in *trans* via small RNAs, via a dominant repressing action (*ARR17* inv. rep: *ARR17* inverted repeats). **(B)** Intriguingly, the same sex gene appears to be a sex-determining gene in a ZW system in white poplar (*P*. *alba*). Copies of the gene are found in the SDR of this species, and its absence from the Z chromosome (dashed gray box) leads to recessive loss of female and activation of male function.

The diversity of sexual systems in plants indicates various mechanisms, including sex determination via a single sex switch (female ↔ male). Experimental support for such switches in dioecious plant species was provided by Akagi et al. (2014) in the Caucasian persimmon and by Müller et al. (2020) in *Populus* spp. (Figure 2).

The genus *Diospyros* contains approximately 475 species of which all are dioecious (Renner, 2016). A male-specific sex-determining gene was described in the Caucasian persimmon (*Diospyros lotus*), that presents a male heterogametic system (XY) and a small SDR (Akagi et al., 2014). The male-specific *Oppressor of MeGI* (*OGI*) produces an RNA hairpin and, through a small RNA-based mechanism apparently causing DNA methylation, represses the autosomal *MALE GROWTH INHIBITOR* (*MeGI*) gene, allowing male development (Figure 2A and Table 1). Since no transformation protocol is available for *D. lotus*, the functional characterization of *OGI* and *MeGI* was performed in *Nicotiana benthamiana, N*. *tabacum*, and *A. thaliana*, showing that the overexpression of *OGI* suppressed the expression of *MeGI* and the overexpression of *MeGI* inhibited male flower function (Akagi et al., 2014). *MeGI* is a homolog of the *HOMEODOMAIN LEUCINE ZIPPER PROTEIN* (*Vrs1*), a gene from barley (*Hordeum vulgare*) (Komatsuda et al., 2007) that, when mutated, produces a group of three fertile flowers instead of a single central one. In persimmons, male flowers are composed by stamens, organized in groups of three, and non-developed carpels, suggesting the strong influence of this gene on sex determination and flower development (Akagi et al., 2016). Although experimental data only confirm the influence of the *OGI*/*MeGI* module on androecia and not gynoecia development, the *D. lotus* system has been suggested to function via the single *OGI*/*MeGI* sex switch. Recently, a gene network analysis identified the pathways regulating male and female sexual organ development. Furthermore, expression levels of cytokinin-related genes during gynoecium differentiation are correlated with *MeGI* expression levels (Yang et al., 2019). This suggests the cytokinin signaling pathway could play a role in the gynoecium differentiation in persimmon flowers.

**TABLE 1:** Candidate genes for sex determination with or without functional validation in different dioecious plant species.

**Species**	**Original gene name**	**Function of ortholog/original name**	**Species of ortholog**	**Reference**
*Actinidia deliciosa*	Shy girl	*ARABIDOPSIS RESPONSE REGULATOR 24 (ARR24)*	*Arabidopsis thaliana*	Akagi et al., 2018, 2019
	Friendly boy	*FAS1 DOMAIN PROTEIN (FAS1)*	*Nicotiana benthamiana*	
*Asparagus officinalis*	*SOFF*	*DOMAIN OF UNKNOWN FUNCTION 247 (DUF247)*	*Medicago truncatula*	Harkess et al., 2017, 2020
	*aspTDF1*	*DEFECTIVE IN TAPETAL DEVELOPMENT AND FUNCTION 1 (TDF1)*	*Arabidopsis thaliana*	
*Diospyros lotus*	*MeGI*	*HOMEODOMAIN LEUCINE ZIPPER CLASS (HD-Zip I) PROTEIN (HB40)*	*Hordeum vulgare*	Akagi et al., 2014
*Fragaria virginiana, Fragaria chiloensis*	*GMEW*	*GDP-MANNOSE 3,5-EPIMERASE 2 (GMEW)*	Not applicable	Tennessen et al., 2018
	*RPP0W*	*60S ACIDIC RIBOSOMAL PROTEIN P0 (RPP0W)*	Not applicable	
*Ginkgo biloba*	Gb_15883	*ARABIDOPSIS RESPONSE REGULATOR 12 (ARR12)*	*Arabidopsis thaliana*	Zhang et al., 2019
	Gb_15884	*ARABIDOPSIS RESPONSE REGULATOR 2 (ARR2)*	*Arabidopsis thaliana*	
	Gb_15885	*EARLY FLOWERING 6 (ELF6)*	*Arabidopsis thaliana*	
	Gb_15886	*BRASSINOSTEROID-RELATED ACYLTRANSFERASE1 (BAT 1)*	*Arabidopsis thaliana*	
	Gb_28587	*AGAMOUS-LIKE 6 (AGL6)*	*Arabidopsis thaliana*	
*Nepenthes gracilis, Nepenthes rafflesiana, Nepenthes pervillei*	*DYT1*	*DYSFUNCTIONAL TAPETUM 1 (DYT1)*	*Arabidopsis thaliana*	Scharmann et al., 2019
	*SEP 1*	*SEPALLATA1 (SEP1)*	*Arabidopsis thaliana*	
*Phoenix dactylifera*	*CYP*	*CYTOCHROME P450 HYDROXYLASE (CYP703A3)*	*Oryza sativa*	Torres et al., 2018
	*GPAT3-like*	*GLYCEROL-3-PHOSPHATE ACYLTRANSFERASE 3-LIKE (GPAT3)*	*Oryza sativa*	
	*LOG*	*LONELY GUY (LOG)*	*Oryza sativa*	
*Populus tremula, Populus alba*	*ARR17*	*ARABIDOPSIS RESPONSE REGULATOR 17 (ARR17)*	*Arabidopsis thaliana*	Müller et al., 2020
*Silene latifolia*	*SlAP3*	*APETALA 3 (AP3)*	*Arabidopsis thaliana*	Cegan et al., 2010
	*SlSTM*	*SHOOT MERISTEMLESS (STM)*	*Arabidopsis thaliana*	Zluvova et al., 2006
	*SlCUC*	*CUP-SHAPED COTYLEDON 1 and 2 (CUC1/CUC2)*	*Arabidopsis thaliana*	
*Salix purpurea*	*SpRR9* (*ARR17*)	*ARABIDOPSIS RESPONSE REGULATOR 17 (ARR17)*	*Arabidopsis thaliana*	Zhou et al., 2020a
*Vitis vinifera*	*VviINP1*	*INAPERTURATE POLLEN 1 (INP1)*	*Arabidopsis thaliana*	Massonnet et al., 2020
	*APRT3*	*ADENINEPHOSPHORIBOSYL TRANSFERASE (APT3)*	*Arabidopsis thaliana*	
	*VviYABBY3*	*YABBY DOMAIN CLASS TRANSCRIPTION FACTOR (YAB1)*	*Arabidopsis thaliana*	

In the genus *Populus*, the SDR was identified on chromosome 19 in different species (Gaudet et al., 2008; Paolucci et al., 2010; Kersten et al., 2014; Geraldes et al., 2015). Genome-wide association studies (GWAS) revealed a homolog of the *A. thaliana* gene pair *ARABIDOPSIS RESPONSE REGULATOR 16* (*ARR16*)/*ARR17*, which was named *ARR17*, as a strong candidate for sex determination in the closely related balsam poplars *Populus balsamifera* and *Populus trichocarpa* (Geraldes et al., 2015; McKown et al., 2017). Further analysis identified partial duplicates of *ARR17* in the male-specific region of the Y chromosome (MSY) (Müller et al., 2020). Notably, these duplicates are present in aspens and balsam poplars, which represent two different sections of the genus, suggesting the possibility of a shared mechanism of sex determination. Despite these commonalities, phylogenetic analysis indicated that the sex-linked *ARR17* duplicates evolved independently (Müller et al., 2020; Zhou et al., 2020b). Long read sequencing showed that the partial duplicates are arranged as inverted repeats, giving rise to small RNAs and apparently causing DNA methylation and silencing of the *ARR17* gene, reminiscent of the *OGI*/*MeGI* system of persimmon (Figure 2A; Bräutigam and Cronk, 2018; Müller et al., 2020). Most importantly, the functionality of *ARR17* as a sex switch was demonstrated by CRISPR/Cas9-mediated *arr17* knockout in early flowering aspen lines, reverting females to fully functional males (Müller et al., 2020). This complete sex reversal, rather than a reversion to hermaphrodites or neuters, demonstrates that *ARR17* functions as a single-gene sex switch.

Interestingly, white poplars (*Populus alba*) present a female heterogametic system (ZW) (Paolucci et al., 2010). Long read sequencing and *de novo* assembly of a female white poplar identified a W chromosome-specific contig with three complete copies of *ARR17* (Müller et al., 2020). Male white poplars do not carry any *ARR17* sequences in their genome. Sex determination in white poplars thus appears to be based on a simple presence/absence mutation of *ARR17* (Figure 2B). Intriguingly, the single-gene-based mechanism of dioecy provides a simple and elegant means of changing the heterogametic system. The transition from a dominant suppressor, which acts in trans, to a recessive presence/absence mutation, which acts in *cis*, leads to the switch in the heterogametic system from XY to ZW (Figure 2; Müller et al., 2020).

### Unified Model of Sex Determination

**FIGURE 3:**
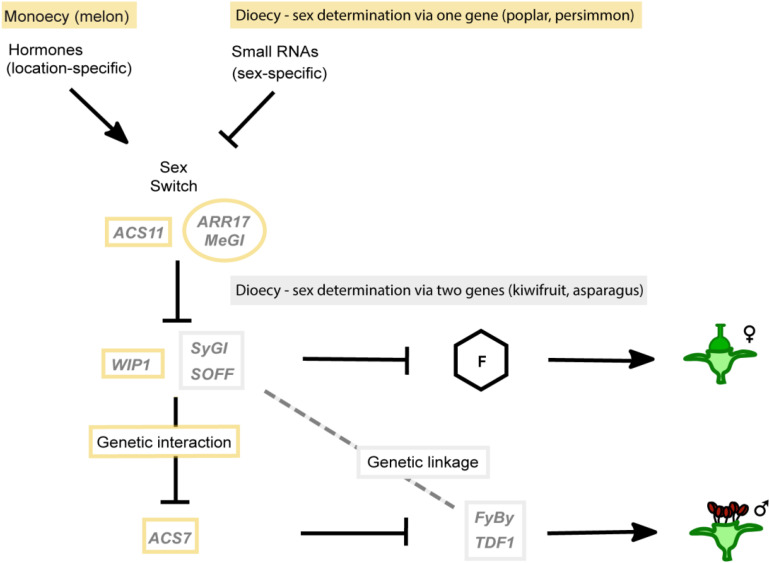
Unified model of sex determination in monoecious and dioecious plants. All experimentally proven systems of sex determination can be combined into a single genetic network comprising female and male suppressors, female and male promoters and high-level sex switches. In the “sex switch systems,” i.e., monoecy and sex determination via one gene, indicated in yellow, an epistatic genetic interaction connects the development of female and male flowers making them in principle mutually exclusive. In sex determination via two genes (“gynodioecy model”) indicated in gray, instead of a genetic interaction, the linkage between the female suppressor (*SOFF* in asparagus and *SyGI* in kiwifruit) and a male promoter (*TDF1* in asparagus and *FyBy* in kiwifruit) are essential for connecting female and male flower development. Female and male floral organs are controlled independently, therefore requiring genetic linkage to avoid hermaphrodites or asexual neuters. Female promoters, represented by “F,” have not been identified for any of the studied species to date.

Interestingly, all currently known molecular mechanisms underlying sex determination can be represented in a single genetic network consisting of suppressors and promoters of femaleness and maleness connected either via genetic linkage or an epistatic genetic interaction (Figure 3). For monoecious species and dioecious species that determine sex via one gene, an additional high-level sex switch is needed. This switch (*ASC11* in melon, *ARR17* in poplar, and *MeGI* in persimmon) represses a female suppressor (*WIP1* in melon) that in turn represses a male suppressor (*ACS7* in melon) by genetic interaction. In sex-determining systems via two genes, there is no genetic interaction, but the female suppressor and male promoter are linked into the sex-determining region. This unified model of plant sex determination emphasizes the differences and commonalities of the different systems and highlights the reason why one might hypothesize that single-gene sex-determining systems could be common in dioecious species that evolved via monoecy.

### Experimental Findings in Selected Other Species

Sex determination has been studied in many other plant species. In the following paragraphs we review studies that provide robust data on sex-determining regions and potential candidate genes.

The early diverged lineage of land plants represented by *Ginkgo biloba* exhibits an ancient origin of dioecy. Recent data indicate a male heterogametic system (XY) (Zhang et al., 2019). The species has a large (10.61 Gb) and repetitive genome (Guan et al., 2016), which was assembled into 12 chromosomes with approximately 9 Gb (Zhang et al., 2019). A large region (∼4.6 Mb) on chromosome 2 was identified as the SDR. Within this region, 16 protein-coding genes were found, and from those, five were suggested as potential sex-determining genes mainly due to their connection to previously reported functions in sex determination in other species: homologs of *RESPONSE REGULATOR 12* (*RR12*) and *RESPONSE REGULATOR 2* (*RR2*) (Gb_15883 and Gb_15884, respectively), both previously reported to be related to cytokinin and sex determination in kiwifruit (Akagi et al., 2018); a homolog of *EARLY FLOWERING 6* (*ELF6*) (Gb_15885), which encodes a H3K4 demethylase involved in the regulation of flowering time; a homolog of *BRASSINOSTEROID-RELATED ACYLTRANSFERASE 1* (*BAT1*) (Gb_15886), which regulates sex determination in maize (Hartwig et al., 2011); and a homolog of *AGAMOUS-like 6* (*AGL6*) (Gb_28587), reported to specify floral organ identity in rice (Li et al., 2010). To further explore sex determination in *G. biloba*, data of gene expression in different developmental stages should reveal clues to advance the investigations on this non-model gymnosperm species.

*Fragaria* spp. may display various sexual systems (e.g., hermaphrodite, gynodioecy, subdioecy, and dioecy) across the species providing a great opportunity for new insights into the evolution of unisexuality in plants. The dioecious octoploid wild strawberry (*Fragaria virginiana*) was characterized as having homomorphic sex chromosomes with a female heterogametic system ZW (Spigler et al., 2008; Goldberg et al., 2010; Tennessen et al., 2016). Strikingly, a small female-specific sequence (13 kb) was recognized as the SDR “cassette,” which is located at different genomic positions in three related *Fragaria* species (Wei et al., 2017; Tennessen et al., 2018). The translocation of the SDR cassette demonstrates a possible way of sex chromosome turnover (Wei et al., 2017; Tennessen et al., 2018). Interestingly, only two protein-coding genes, *GMEW* (*GDP-mannose 3,5-epimerase 2, GME)* and *RPP0W (60S acidic ribosomal protein P0, RPP0*), were found in this “cassette.” Nevertheless, it remains unclear how these candidate genes act in sex determination (Tennessen et al., 2018). Moreover, the SDR “cassette” might only control male function, while female function is controlled by a second locus (Spigler et al., 2008).

In willow (*Salix* spp.), the SDR was identified on chromosome 15 with female heterogamety (ZW) in *Salix viminalis* (Pucholt et al., 2015), *Salix suchowensis* (Hou et al., 2015; Chen et al., 2016), *Salix purpurea* (Zhou et al., 2018), and *Salix triandra* (Li et al., 2020). A recent study revealed large palindromic structures on the W chromosome of *S. purpurea* and an ortholog of *ARR17* (*Salix purpurea RESPONSE REGULATOR* 9, *SpRR9*) was suggested as a strong candidate gene for sex determination (Zhou et al., 2020a). In contrast, in another species, *Salix nigra*, a relatively small SDR (∼2 Mb) was identified on chromosome 7 presenting a male heterogametic system (XY) (Sanderson et al., 2020). The underlying mechanisms for sex determination in *Salix* remain unclear; however, there is a possibility of a shared mechanism of sex determination despite the dynamic turnover of sex chromosomes in Salicaceae species.

Sex determination has also been investigated in *Nepenthes* pitcher plants (Scharmann et al., 2019). The species of this genus are all dioecious and carnivorous. Based on wild populations of males and females of three different species (*Nepenthes pervillei, Nepenthes gracilis*, and *Nepenthes rafflesiana*), data supporting a male heterogametic system (XY) were presented. Two expressed sex-linked genes were identified: the homologs of the *A. thaliana* genes *DYSFUNCTIONAL TAPETUM 1* (*DYT1*) and *SEPALLATA 1* (*SEP1*)*;* The first with important role in tapetum development and pollen fertility and the second as a regulator of floral organ identity. The *DYT1* gene functions in the tapetum, similar to the male-promoting genes in kiwifruit and asparagus. This opens the possibility of sex determination via two genes, where *DYT1* could function as the male-promoting factor.

*Silene latifolia*, (white campion), is a widely studied species and a model for studying sex chromosome evolution. It presents heteromorphic sex chromosomes and a male heterogametic system (XY) (Blackburn, 1923; Bernasconi et al., 2009; Kejnovsky and Vyskot, 2010; Muyle et al., 2012). Over the years, several genes have been discussed as potential sex determining factors: *S. latifolia X/Y-gene 1* (*SIX/Y1*), encoding a WD-repeat protein and likely involved in cell proliferation and *SlX/Y4*, encoding a fructose-2,6-bisphosphatase (Atanassov et al., 2001); the floral organ identity gene *APETALA 3* (*SlAP3*) (Cegan et al., 2010), which is specifically involved in the development of androecia, and orthologs of *SHOOT MERISTEMLESS* (*STM*) (named *SlSTM1* and *SlSTM2*) and *CUP-SHAPED COTYLEDON 1* (*CUC1*) and *CUC*2 (denoted as *SlCUC*) (Zluvova et al., 2006), both activators of cytokinin biosynthesis (Yang et al., 2019). The function of either of these genes remains to be tested. Recent deletion mapping in *Silene* (Kazama et al., 2016) improved the locations of the sex-determining loci on the Y chromosome and could help to identify candidate sex-determining genes for further testing.

For details including other species, Supplementary Table 1 presents a more complete list with respective references.

## Summary of Sex-Determining Genes in Different Dioecious Plant Species

Although there are still numerous species of which the molecular and physiological mechanisms of sex determination remain elusive, the recent progress described above is remarkable and finally allows first comparative analyses. Despite multiple origins of dioecy, there might still be similar genes and pathways employed repeatedly for the independent evolution of dioecy. Such similarities can only be identified now that several systems can be analyzed together.

**FIGURE 4:**
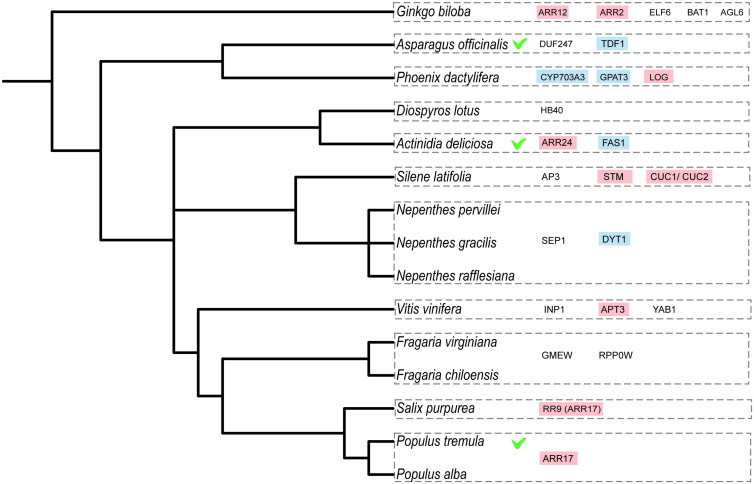
Evolutionary relationship of dioecious species with known sex-determining genes or strong candidate genes for sex determination (based on Table 1). Marked with a green tick the species with genes that were functionally validated in the species, in pink the cytokinin-related genes and in blue the tapetum-related genes. The phylogenetic tree was built using phyloT v2: a tree generator (based on NCBI taxonomy; https://phylot.biobyte.de/).

Interestingly, a first tentative analysis revealed a remarkably high number of genes involved in cytokinin signaling (Table 1 and Figure 4). Cytokinin is a plant hormone known to be important for gynoecium formation (Marsch-Martinez et al., 2012). This also becomes evident by exogenous application of cytokinin, which can initiate carpel development in several species, including grapes (Wang et al., 2013), persimmon (Yonemori et al., 1993), and kiwifruit (Akagi et al., 2018). In this regard it is noteworthy that in monoecious species plant hormones play an essential role in sex determination (West and Golenberg, 2018). For instance, in maize, flower development is connected to the jasmonic acid signaling pathway (Acosta et al., 2009), whereas in melon, ethylene appears to be the major player (Boualem et al., 2015). In dioecious species, hormones appear to influence sexual differentiation as well. For example, the sex switch *ARR17* in poplar is a type-A cytokinin response regulator (Müller et al., 2020). Moreover, a network analysis identified the cytokinin pathway as an important component of flower development in persimmon (Yang et al., 2019). Interestingly, in species that determine sex via two genes, several of the genes encoding suppressors of female development (*Su*^*F*^) are involved in cytokinin signaling as well. These genes include, among others, *SyGI* in kiwifruit, which encodes a cytokinin response regulator (Akagi et al., 2018), the candidate gene *LOG* in date palm (Torres et al., 2018), which encodes a cytokinin-activating enzyme, and the candidate gene *APRT3* in grapes (Badouin et al., 2020), which encodes an enzyme involved in the inactivation of cytokinin. Finally, two cytokinin response regulators are located in the SDR of Ginkgo (Zhang et al., 2019). Together these data strongly suggest an important role of cytokinin signaling for sex determination (Figure 4). Despite the single-gene-based mechanism of sex determination in persimmon, it might be interesting to note that a cytokinin response regulator is located in the persimmon SDR as well (Akagi et al., 2014). Notably, the unified model (Figure 3) already indicates that the female suppressors may be closer to the genetic pathways found in monoecious species, thus explaining the potential excess of hormone-related genes.

The male-promoting factors, on the other hand, appear to include several genes that act in tapetum formation and are thus much more directly involved in floral organ development (Table 1 and Figure 4), which again appears consistent with the unified model of sex determination (Figure 3). The genes encoding the male promoters in kiwifruit and asparagus, i.e., *FyBy* and *TDF1*, respectively, and the candidate genes *CYP* and *GPAT3* in date palm and *DYT1* in *Nepenthes* all function in the tapetum potentially influencing male fertility. The putative male promoter in grapes (*VviINP1*) functions in pollen development and is thus also very directly involved in floral organ functioning, rather than in more general hormone signaling.

Despite the small number of candidate sex-determining genes identified to date, there appear to be some overarching patterns. In species that determine sex via two genes, the male-promoting factors seem to act specifically in the androecium to allow functional male floral organ development. From the other candidate genes, including female suppressors and sex switches, several seem to function in similar hormone response pathways. The many cytokinin-related genes in different dioecious species (Table 1 and Figure 4), suggest that cytokinin signaling may be especially important in the regulation of sex determination predestinating it for the evolution of dioecy. It will be exciting to extend the comparison with further sex-determining genes in the future to assess whether these first generalizations remain valid.

## Conclusion and Outlook

Dioecy has evolved numerous times independently. Despite elegant theoretical models for the evolution of dioecy (Charlesworth and Charlesworth, 1978; Renner, 2016), only recently, powerful experimental work is providing empirical data for further assessing different possible trajectories (Harkess et al., 2017, 2020; Akagi et al., 2019; Müller et al., 2020). These data highlight the diversity of sex-determining mechanisms and emphasize the need for considering more than just one theoretical model.

A first tentative comparative analysis of sex-determining and candidate genes in different dioecious species suggests that similar genes and pathways may be employed repeatedly for the independent evolution of dioecy. Cytokinin-related genes appear to be important in sex determination of several dioecious species, irrespective of whether sex is determined via one or two genes. Moreover, tapetum-related genes were identified as male-promoting factors in several two-gene systems.

The expanding number of studies related to sex chromosome evolution and sex-determining systems in crop plants may contribute to enhancing their agricultural value. Studies in model systems provide further important biological insights into chromosome evolution and the molecular mechanisms of flower development. Novel molecular techniques such as long read sequencing, transformation protocols or gene editing approaches are rapidly becoming available to support the identification of the sex-determining genes and the underlying genetic mechanisms leading to the evolution of dioecy. Thus, it is likely that several sex-determining systems will be resolved in the next couple of years. Furthermore, the pathway connecting these high-level regulators to floral phenotype is largely unknown, and work in this area is urgently required if we are to fully understand dioecy (Feng et al., 2020; Cronk and Müller, 2020). These data will allow exciting further generalizations and improve our understanding of the molecular control and the evolution of dioecy in flowering plants.

## Author Contributions

AL: original draft. BK, MF, and NM: inputs and revision. All authors contributed to the article and approved the submitted version.

## Conflict of Interest

The authors declare that the research was conducted in the absence of any commercial or financial relationships that could be construed as a potential conflict of interest.
