# Supplementary material for: The Diversity and Dynamics of Sex Determination in Dioecious Plants
Source: Front Plant Sci. 2021 Jan 15;11:580488. doi: 10.3389/fpls.2020.580488 (PMC7843427; doi:10.3389/fpls.2020.580488)
Supplement: Supplementary file 1 [file Table_1.DOCX]

Supplementary Table 1: Dioecious plant species with cytogenetic and/or molecular evidence for the presence of sex chromosomes and heterogametic sex (UV, XY or ZW) in a phylogenetic perspective.

| **Phylum** | **Order** | **Family** | **Species** | **Sexual system** | | **Reference** |
| --- | --- | --- | --- | --- | --- | --- |
|  |  |  |  | **F** | **M** |  |
| Bryophyta  Mosses | Pseudoditrichales | Ditrichaceae | *Ceratodon purpureus* | U | V | [1], [2] |
| Bryophyta  Liverworts | Marchantiales | Marchantiaeceae | *Marchantia polymorpha* | U | V | [3]–[6] |
|  |  |  | *Marchantia inflexa* | U | V | [7] |
|  | Porellales | Frullaniaceae | *Frullania dilatata* | U | V | [8], [9] |
|  | Sphaerocarpales | Sphaerocarpaceae | *Sphaerocarpos donnellii* | U | V | [10], [11] |
|  |  |  | *Sphaerocarpos texanus* | U | V |  |
| Gymnosperms | Cycadales | Cycadaceae | *Cycas revoluta* | XX | XY | [12], [13] |
|  |  |  | *Cycas pectinata* | XX | XY | [14] |
|  | Ginkgoales | Ginkoaceae | *Ginkgo biloba* | XX | XY | [15], [16] |
|  | Araucariales | Podocarpaceae | *Podocarpus macrophyllus* | X_1_X_2_ | YX_1_X_2_ | [17], [18] |
|  |  |  | *Podocarpus longefoliolatus* | X_1_X_2_ | YX_1_X_2_ |  |
|  |  |  | *Podocarpus elatus* | X_1_X_2_ | YX_1_X_2_ |  |
| Angiosperms  Angiosperms  Angiosperms | Ericales | Actinidiaceae | *Actinidia chinensis* | XX | XY | [19]–[22] |
|  |  |  | *Actinidia deliciosa* | XX | XY |  |
|  |  | Ebenaceae | *Diospyros lotus* | XX | XY | [23] |
|  | Caryophyllales | Amarantheaceae | *Amaranthus tuberculatus* | XX | XY | [24] |
|  |  | Caryophyllaceae | *Silene otites* | ZW | ZZ | [25]–[27] |
|  |  |  | *Silene pseudotites* | XX | XY | [25], [27] |
|  |  |  | *Silene colpophylla* | XX | XY | [28], [29] |
|  |  |  | *Silene latifolia* | XX | XY | [30]–[33] |
|  |  |  | *Silene dioica* | XX | XY |  |
|  |  |  | *Silene diclinis* | XX | XY | [34] |
|  |  | Chenopodiaceae | *Spinacia oleracea* | XX | XY | [35]–[37] |
|  |  | Polygonaceae | *Rumex acetosa* | XX | XY_1_Y_2_ | [38], [39] |
|  |  |  | *Rumex acetosella* | XX | XY | [38] – [41] |
|  |  |  | *Rumex graminifolius* | XX | XY | [41] |
|  |  |  | *Rumex hastatulus* | XX | XY | [42], [43] |
|  |  |  | *Rumex papillaris* | XX | XY_1_Y_2_ | [44] |
|  |  |  | *Rumex paucifolius* | XX | XY | [45] |
|  |  |  | *Rumex suffruticosus* | XX | XY | [40] |
|  |  | Nepenthaceae | *Nepenthes pervillei* | XX | XY | [46] |
|  |  |  | *Nepenthes gracilis* | XX | XY |  |
|  |  |  | *Nepenthes rafflesiana* | XX | XY |  |
|  | Brassicales | Caricaceae | *Carica papaya* | XX | XY | [47]–[49] |
|  |  |  | *Vasconcellea cundinamarcensis* | XX | XY |  |
|  |  |  | *Vasconcellea parviflora* | XX | XY |  |
|  |  |  | *Vasconcellea pulchra* | XX | XY |  |
|  | Rosales | Cananbaceae | *Cannabis sativa* | XX | XY | [50]–[52] |
|  |  |  | *Humulus lupulus* | XX | XY | [53]–[58] |
|  |  |  | *Humulus lupulus var. cordifolius* | X_1_X_1_X_2_X_2_ | X_1_Y_1_X_2_ Y_2_ |  |
|  |  |  | *Humulus japonicus* | XX | XY_1_Y_2_ |  |
|  |  | Rosaceae | *Fragaria virginiana* | ZW | ZZ | [59]–[61] |
|  |  |  | *Fragaria chiloensis* | ZW | ZZ |  |
|  |  |  | *Fragaria moschata* | ZW | ZZ |  |
|  |  | Elagnaceae | *Hippophae rhamnoides* | XX | XY | [62]–[64] |
|  | Curcubitales | Curcubitaceae | *Bryonia dioica* | XX | XY | [65]–[67] |
|  |  |  | *Ecballium elaterium* | XX | XY | [68] |
|  |  |  | *Coccinia grandis* | XX | XY | [69]–[72] |
|  |  |  | *Trichosanthes dioica* | XX | XY | [73]–[76] |
|  |  |  | *Trichosanthes kirilowii* | XX | XY |  |
|  |  |  | *Trichosanthes kirilowii var. japonica* | XX | XY |  |
|  |  |  | *Trichosanthes multiloba* | XX | XY |  |
|  |  |  | *Trichosanthes ovigera* | XX | XY |  |
|  |  |  | *Trichosanthes cucumeroides* | XX | XY |  |
|  |  | Datiscaceae | *Datisca cannabina* | ZW | ZZ | [77] |
|  | Dioscoreales | Dioscoreaceae | *Dioscorea rotundata* | ZW | ZZ | [78], [79] |
|  |  |  | *Dioscorea deltoidea* | ZW | ZZ | [80] |
|  |  |  | *Dioscorea tokoro* | XX | XY | [81], [82] |
|  |  |  | *Dioscorea alata* | XX | XY | [83] |
|  | Fagales | Myricaceae | *Morella rubra* | XX | XY | [84] |
|  | Sapyndales | Anacardiaceae | *Pistacia vera* | ZW | ZZ | [85]–[88] |
|  | Arecales | Arecaceae | *Phoenix dactylifera* | XX | XY | [89]–[94] |
|  |  |  | *Phoenix reclinata* | XX | XY |  |
|  |  |  | *Phoenix sylvestris* | XX | XY |  |
|  |  |  | *Phoenix atlantica* | XX | XY |  |
|  |  |  | *Phoenix canariensis* | XX | XY |  |
|  |  |  | *Phoenix roebelenii* | XX | XY |  |
|  |  |  | *Phoenix rupicola* | XX | XY |  |
|  |  |  | *Phoenix acaulis* | XX | XY |  |
|  | Aspargales | Asparagaceae | *Asparagus officinalis* | XX | XY | [95] |
|  | Pentapetalae | Santalaceae | *Viscum fischeri* | X_1_X_2_X_3_X_4_ | Y_1_Y_2_Y_3_Y_4_ | [96] |
|  | Malpighiales | Salicaceae | *Populus tremula* | XX | XY | [97] – [102] |
|  |  |  | *Populus trichocarpa* | XX | XY |  |
|  |  |  | *Populus balsamifera* | XX | XY |  |
|  |  |  | *Populus tremuloides* | XX | XY |  |
|  |  |  | *Populus nigra* | XX | XY |  |
|  |  |  | *Populus deltoides* | XX | XY |  |
|  |  |  | *Populus alba* | ZW | ZZ |  |
|  |  |  | *Salix purpurea* | ZW | ZZ | [103] |
|  |  |  | *Salix viminalis* | ZW | ZZ | [104] |
|  |  |  | *Salix suchowensis* | ZW | ZZ | [105] |
|  |  |  | *Salix triandra* | ZW | ZZ | [106] |
|  |  |  | *Salix nigra* | XX | XY | [107] |
|  |  | Euphorbiaceae | *Mercurialis annua* | XX | XY | [108]–[110] |
|  | Vitales | Vitaceae | *Vitis vinifera* | XX | XY | [111], [112] |

## References

[1] McDaniel, S.F., Willis, J.H., Shaw, A.J. (2007). A linkage map reveals a complex basis for segregation distortion in an interpopulation cross in the moss *Ceratodon purpureus*. *Genetics*. 176 (4):2489-2500. https://doi.org/10.1534/genetics.107.075424

[2] McDaniel,S.F., Neubig, K.M., Payton, A.C., Quatrano, R.S., Cove, D.J. (2013). Recent gene-gapture on the UV sex chromosomes of the Moss C*eratodon purpureus*. *Evolution*. 67 (10):2811-2822. https://doi.org/10.1111/evo.12165

[3] Okada, S., Fujisawa, M., Sone, T., Nakayama, S., Nishiyama, R., Takenaka, M. et al*.* (2000). Construction of male and female PAC genomic libraries suitable for identification of Y-chromosome-specific clones from the liverwort, *Marchantia polymorpha*. *The* *Plant Journal.* 24 (3): 421-428. https://doi.org/10.1046/j.1365-313x.2000.00882.x

[4] Okada, S., Sone, T., Fujisawa, M., Nakayama, S., Takenaka, M., Ishizaki, K. et al. (2001).The Y chromosome in the liverwort *Marchantia polymorpha* has accumulated unique repeat sequences harboring a male-specific gene,” *Proceedings of the Nationa. Academy of Sciences of the United States of America*. 98 (16): 9454-9459. https://doi.org/10.1073/pnas.171304798

[5] Fujisawa M., Hayashi, K., Nishio, T., Bando, T., Okada, S., Yamato, K.T. et al. (2001). Isolation of X and Y chromosome-specific DNA markers from a liverwort, *Marchantia polymorpha*, by representational difference analysis. *Genetics*. 159 (3):981-985.

[6] Yamato, K.T., Ishizaki, K., Fujisawa, M., Okada, S., Nakayama, S., Fujishita, M. et al. (2007). Gene organization of the liverwort Y chromosome reveals distinct sex chromosome evolution in a haploid system. *Proceedings of the Nationa. Academy of Sciences of the United States of America*. 104 (15):6472-6477.

[7] Marks, R.A., Smith, J.J., Cronk, Q., Grassa, C.J., McLetchie, D.N. (2019). Genome of the tropical plant *Marchantia inflexa*: implications for sex chromosome evolution and dehydration tolerance. *Scientific Reports*. 9 (1):8722. https://doi.org/10.1038/s41598-019-45039-9

[8] Temsch, E.M., and Greilhuber, J. (2010). Genome size in Dipsacaceae and *Morina longifolia* (Morinaceae). *Plant Systematics and Evolution*. 289 (1-2):45-56. https://doi.org/10.1007/s00606-010-0330-1

[9] Renner, S.S., Heinrichs, J., Sousa, A. (2017). The sex chromosomes of bryophytes: Recent insights, open questions, and reinvestigations of *Frullania dilatata* and *Plagiochila asplenioides*. *Journal of Systematics and Evolution.* 55 (4): 333–339. https://doi.org/10.1111/jse.12266

[10] Allen, C.E. (1917). A chromosome difference correlated with sex differences in *Sphærocarpos*. *Science*.46 (1193): 466-467. https://doi.org/10.1126/science.46.1193.466

[11] McLetchie D.N., and Collins, A.L., (2001). Identification of DNA Regions Specific to the X and Y Chromosomes in *Sphaerocarpos texanus*. *Bryologist*. 104 (4): 543-547. https://doi.org/10.1639/0007-2745(2001)104[0543:IODRST]2.0.CO;2

[12] Segawa, M., Kishi, S., Tatuno, S. (1971). Sex chromosomes of *Cycas revoluta*. *Japanese Journal of Genetics*. 46 (1): 33–39. https://doi.org/10.1266/jjg.46.33

[13] Gorelick, R., and Osborne, R. (2007). Evolution of Dioecy and Sex Chromosomes in Cycads. In: *Reproductive Biology of Plants.* Johri, B.M. and Srivastava, P.S. (Eds). 97:326-334.

[14] Abraham, A., and Mathew, P.M., (1962). Cytological Studies in the Cycads: Sex Chromosomes in *Cycas*,” *Annals of Botany.* 26 (2):261–266. https://doi.org/10.1093/oxfordjournals.aob.a083792

[15] Lee, C.L. (1954). Sex Chromosomes in *Ginkgo biloba.* *American Journal of Botany*.41 (7):545-549. https://doi.org/10.1002/j.1537-2197.1954.tb14376.x

[16] Zhang H., Zhang, R., Yang, X., Gu, K.J., Chen, W., Chang., et al. (2019). Recent origin of an XX/XY sex-determination system in the ancient plant lineage *Ginkgo biloba*. *BioRxiv* [*Preprint*]. https://doi.org/10.1101/517946

[17] Hizume, M., Shiraishi, H., Tanaka, A. (1988). A cytological study of *Podocarpus* *macrophyllus* with special reference to sex chromosomes. *Japanese Journal of Genetics.*63 (5):413-423. https://doi.org/ 10.1266/jjg.63.413

[18] Hair, J.B., and Beuzenberg, E.J. (1958). Chromosomal evolution in the Podocarpaceae. *Nature*. 181: 1584-1586. https://doi.org/10.1038/1811584a0

[19] Fraser, L.G., Tsang, G.K., Datson, P.M., De Silva, H.N., Harvey, C.F., Gill, G.P., Crowhurst, R.N., McNeilage, M.A. (2009). A gene-rich linkage map in the dioecious species *Actinidia chinensis* (kiwifruit) reveals putative X/Y sex-determining chromosomes. *BMC Genomics*. 10, 102. https://doi.org/10.1186/1471-2164-10-102

[20] Gill, G.P., Harvey, C.F., Gardner, R.C., Fraser, L.G. (1988). Development of sex-linked PCR markers for gender identification in *Actinidia*. *Theoretical and Applied Genetics*. 97: 439-445. https://doi.org/10.1007/s001220050914

[21] Testolin, R., Huang, W.G., Lain, O., Messina, R., Vecchione, A., Cipriani, G. (2001). A kiwifruit (*Actinidia* spp.) linkage map based on microsatellites and integrated with AFLP markers. *Theoretical and Applied Genetics*. 103: 30-36. https://doi.org/10.1007/s00122-001-0555-z

[22] Akagi, T., Henry, I.M., Ohtani, H., Morimoto, T., Beppu, K., Kataoka, I., Tao, R. (2018). A Y-encoded suppressor of feminization arose via lineage-specific duplication of a cytokinin response regulator in kiwifruit. *Plant Cell*. 30 (4):780–795. https://doi.org/10.1105/tpc.17.00787

[23] Akagi, T., Henry, I.M., Kawai, T., Comai, L., Tao, R. (2016). Epigenetic Regulation of the Sex Determination Gene *MeGI* in Polyploid Persimmon. *Plant Cell*. 28 (12): 2905–2915. https://doi.org/10.1105/tpc.16.00532

[24] Murray, M.J. (1940). The Genetics of Sex Determination in the Family Amaranthaceae. *Genetics*. 25 (4): 409-431.

[25] Sansome, F.W. (1938). Sex determination in *Silene otites* and related species. *Journal of Genetics*. 35, 387. https://doi.org/10.1007/BF02982363

[26] Balounova, V., Gogela, R., Cegan, R., Cangren, P., Zluvova, J., Safar, J., Kovacoa, V. et al. (2019). Evolution of sex determination and heterogamety changes in section Otites of the genus *Silene*. *Scientific Reports.* 9, 1045. https://doi.org/10.1038/s41598-018-37412-x

[27] Martin, H., Carpentier, F., Gallina, S., Gode, C., Schmitt, E., Muyle, A., Marais, G.A.B., Touzet, P. (2019)*.* Evolution of young sex chromosomes in two dioecious sister plant species with distinct sex determination systems. *Genome Biology and Evolution.* 11 (2):350–361. https://doi.org/10.1093/gbe/evz001

[28] Moore, R.C., Kozyreva, O., Lebel-Hardenack, S., Siroky, J., Hobza, R., Vyskot, B., Grant, S.R. (2003). Genetic and functional analysis of DD44, a sex-linked gene from the dioecious plant *Silene latifolia*, provides clues to early events in sex chromosome evolution. *Genetics*. 163 (1):321-334.

[29] Mrackova, M., Nicolas, M., Hobza, R., Negrutiu, I., Françoise, M., Widmer, A., Vyskot, B., Janousek, B. (2008). Independent origin of sex chromosomes in two species of the genus *Silene*. *Genetics.* 179 (2):1129–1133. https://doi.org/10.1534/genetics.107.085670

[30] Lebel-Hardenack, S., Hauser, E., Law, T.F., Schmid, J., Grant, S.R. (2002). Mapping of sex determination loci on the white campion (*Silene latifolia*) Y chromosome using amplified fragment length polymorphism. *Genetics*. 160 (2): 717–725.

[31] Blackburn, K.B (1923). Sex chromosomes in plants. *Nature*. 112: 687-688.

[32] Muyle, A., Zemp, N., Deschamps, C., Mousset, S., Widmer, A., Marais, G.A.B. (2012). Rapid de novo evolution of X chromosome dosage compensation in *Silene latifolia*, a plant with young sex chromosomes. *PLoS Biology*. 10 (4):e1001308. https://doi.org/10.1371/journal.pbio.1001308

[33] Kejnovsky, E., and Vyskot, B. (2010). *Silene latifolia*: The classical model to study heteromorphic sex chromosomes. *Cytogenetic and Genome Research.* 129 (1–3):250–262, 2010. https://doi.org/10.1159/000314285

[34] Howell, E.C., Armstrong, S.J., Filatov, D.A. (2009). Evolution of neo-sex chromosomes in *Silene diclinis*. *Genetics*. 182 (4):1109-1115. https://doi.org/10.1534/genetics.109.103580

[35] Ellis, J.R., and Janick, J. (1960). The Chromosomes of *Spinacia oleracea*. *American Journal of Botany*. 47(3):210-214*.* https://doi.org/10.1002/j.1537-2197.1960.tb07115.x

[36] Lan, T., Zhang, S., Liu, B., Li, X., Chen, R., Song, W. (2006). Differentiating sex chromosomes of the dioecious *Spinacia oleracea* L. (spinach) by FISH of 45S rDNA. *Cytogenetic and Genome Research.* 114 (2):175-177. https://doi.org/10.1159/000093335

[37] Deng, C., Qin, R., Gao, J., Cao, Y., Li, S., Gao, W., Lu, L. (2012). Identification of sex chromosome of spinach by physical mapping of 45s rDNAs by FISH. *Caryologia*. 65 (4):322-327. https://doi.org/10.1080/00087114.2012.760879

[38] Kihara, H., add Ono, T. (1923). Cytological Studies on *Rumex* L. *Shokubutsugaku Zasshi*. 37 (435):84-90. https://doi.org/10.15281/jplantres1887.37.435_84

[39] Błocka-Wandas, M., Sliwinska, E., Grabowska-Joachimiak, A., Musial, K., Joachimiak, A.J. (2007). Male gametophyte development and two different DNA classes of pollen grains in *Rumex acetosa* L., a plant with an XX/XY1Y2 sex chromosome system and a female-biased sex ratio. *Sexual Plant Reproduction*. 20 (4):171-180. https://doi.org/ 10.1007/s00497-007-0053-9

[40] Cuñado, N., Navajas-Pérez, R., de la Herrán, R., Rejón, C.R., Rejón, M.R., Santos, J.L., Garrido-Ramos, M.A. (2007). The evolution of sex chromosomes in the genus *Rumex* (Polygonaceae): Identification of a new species with heteromorphic sex chromosomes. *Chromosome Research.* 15:825-833. https://doi.org/10.1007/s10577-007-1166-6

[41] Löve, A. (1944). Cytogenetic studies on *Rumex* subgenus *acetosella*. *Hereditas*. 30 (1-2):1-135. https://doi.org/10.1111/j.1601-5223.1944.tb03303.x

[42] Smith, B.W. (1964). The evolving karyotype of *Rumex hastatulus*. *Evolution.* 18 (1):90-104.

[43] Smith, B.W. (1963). The Mechanism of Sex Determination in *Rumex hastatulus*,” *Genetics*. 48 (10):1265-1288.

[44] Navajas-Pérez, R., Schwarzacher, T., Rejón, M.R., Garrido-Ramos, M.A. (2009). Molecular cytogenetic characterization of *Rumex papillaris*, a dioecious plant with an XX/XY1Y2 sex chromosome system. *Genetica*. 135:87-93. https://doi.org/ 10.1007/s10709-008-9261-y

[45] Smith, B.W. (1969). Evolution of sex-determining mechanisms in *Rumex*. *Chromosomes Today*. 2: 172-182.

[46] Scharmann, M., Grafe, T.U., Metali, F., Widmer, A. (2019). Sex is determined by XY chromosomes across the radiation of dioecious *Nepenthes* pitcher plants. *Evolution Letters.* 3 (6):586–597. https://doi.org/10.1002/evl3.142

[47] Yu, Q., Hou, S., Feltus, F.A., Jones, M.R., Murray, J.E., Veatch, O. et al*.* (2008). Low X/Y divergence in four pairs of papaya sex-linked genes. *Plant Journal.* 53 (1):124-132. https://doi.org/10.1111/j.1365-313X.2007.03329.x

[48] Liu, Z., Moore, P.H., Ma, H., Ackerman, C.M., Ragiba. M., Yu, Q. et al. (2004). A primitive Y chromosome in papaya marks incipient sex chromosome evolution. *Nature*. 427:348-352. https://doi.org/10.1038/nature02228

[49] Iovene, M., Yu, Q., Ming, R., Jiang, J. (2014). Evidence for emergence of sex-determining gene(s) in a centromeric region in *Vasconcellea parviflora.* *Genetics*. 199 (2):413-421. https://doi.org/10.1534/genetics.114.173021

[50] Sakamoto, K., Akiyama, Y., Fukui, K., Kamada, H., Satoh, S. (1998). Characterization; Genome sizes and morphology of sex chromosomes in hemp (*Cannabis sativa* L.). *Cytologia.* 63 (4):459-464. https://doi.org/10.1508/cytologia.63.459

[51] Divashuk, M.G., Alexandrov, O.S., Razumova, O.V., Kirov, I.V., Karlov, G.I. (2014). Molecular cytogenetic characterization of the dioecious *Cannabis sativa* with an XY chromosome sex determination system. *PLoS ONE*. 9 (1): 1–7. https://doi.org/10.1371/journal.pone.0085118

[52] Prentout, D., Razumova, O., Rhone, B., Badouin, H., Henri, H., Feng, C., Käfer, J., Karlov, G., Marais, G.A.B. (2020). An efficient RNA-seq-based segretation analysis identifies the sex chromosomes of Cannabis sativa. Genome Research. 30: 1–9. https://doi.org/10.1101/gr.251207.119

[53] Karlov, G.I., Danilova, T.V., Horlemann, C., Weber, G. (2003). Molecular cytogenetics in hop (*Humulus lupulus* L.) and identification of sex chromosomes by DAPI-banding. *Euphytica*. 132:185-190. https://doi.org/10.1023/A:1024646818324

[54] Polley, A., Seigner, E., Ganal, M.W. (1997). Identification of sex in hop (*Humulus lupulus*) using molecular markers. *Genome*. 40 (3):357-361. https://doi.org/ 10.1139/g97-048

[55] Divashuk, M.G., Alexandrov, O.S., Kroupin, P.Y., Karlov, G.I. (2011). Molecular cytogenetic mapping of *Humulus lupulus* sex chromosomes. *Cytogenetic and Genome Research.* 134 :213-219. https://doi.org/10.1159/000328831

[56] Alexandrov, O.S., Divashuk, M.G., Yakovin, N.A., Karlov, G.I., (2012). Sex chromosome differentiation in *Humulus japonicus* Siebold & Zuccarini, 1846 (Cannabaceae) revealed by fluorescence in situ hybridization of subtelomeric repeat. *Comparative Cytogenetics*. 6 (3):239-247. https://doi.org/ 10.3897/CompCytogen.v6i3.3261

[57] Shephard, H.L., Parker, J.S., Darby, P., Ainsworth, C.C. (2000). Sexual development and sex chromosomes in hop. *New Phytologist*. 148 (3):397-411. https://doi.org/10.1046/j.1469-8137.2000.00771.x

[58] Seefelder, S., Ehrmaier, H., Schweizer, G., Seigner, E., (2000). Male and female genetic linkage map of hops, *Humulus lupulus*. *Plant Breeding.* 119(3):249-255. https://doi.org/10.1046/j.1439-0523.2000.00469.x

[59] Tennessen, J.A., Govindarajulu, R., Liston, A., Ashman, T.L. (2016). Homomorphic ZW chromosomes in a wild strawberry show distinctive recombination heterogeneity but a small sex-determining region. The New Phytologist. 211 (4):1412-1423. https://doi.org/10.1111/nph.13983

[60] Spigler, R.B., Lewers, K.S., Main, D.S., Ashman, T.L. (2008). Genetic mapping of sex determination in a wild strawberry, Fragaria virginiana, reveals earliest form of sex chromosome. Heredity. 101:507–17. https://doi.org/10.1038/hdy.2008.100

[61] Goldberg, M.T., Spigler, R.B., Ashman, T.L. (2010). Comparative genetic mapping points to different sex chromosomes in sibling species of wild strawberry (Fragaria). Genetics. 186 (4): 1425–33. https://doi.org/10.1534/genetics.110.122911

[62] Puterova, J., Razumova, O., Marinek, T., Alexandrov, O., Divashuk, M., Kubat, Z. et al. (2017). Satellite DNA and transposable elements in Seabuckthorn (*Hippophae rhamnoide*s), a dioecious plant with small Y and large x chromosomes. *Genome Biology and Evolution*. 9 (1):197-212. https://doi.org/10.1093/gbe/evw303

[63] Truţǎ, E., Cǎpraru, G., Surdu, Ş., Zamfirache, M.M., Olteanu, Z., Roşu, C.M., Opricǎ, L. (2010). Karyotypic studies in ecotypes of *Hippophae rhamnoides* L. from romania. *Silvae Genetica.* 59 (4):175-182*.* https://doi.org/10.1515/sg-2010-0021

[64] Sharma, A., Zinta, G., Rana, S., Shirko, P. (2010). Molecular identification of sex in *Hippophae rhamnoides* L. using isozyme and RAPD markers. *Forestry Studies in China*. 12: 62-66. https://doi.org/10.1007/s11632-010-0012-7

[65] Oyama, R.K., Volz, S.M., Renner, S.S. (2009). A sex-linked SCAR marker in *Bryonia dioica* (Cucurbitaceae), a dioecious species with XY sex-determination and homomorphic sex chromosomes. *Journal of Evolutionary Biology.* 22 (1): 214-224. https://doi.org/10.1111/j.1420-9101.2008.01641.x

[66] Oyama, R.K., Silber, M.V., Renner, S.S. (2010). A specific insertion of a solo-LTR characterizes the Y-chromosome of *Bryonia dioica* (Cucurbitaceae). *BMC Research Notes*. 3, 166. https://doi.org/10.1186/1756-0500-3-166

[67] Volz, S.M., and Renner, S.S. (2008). Hybridization, polyploidy, and evolutionary transitions between monoecy and dioecy in *Bryonia* (Cucurbitaceae). *American Journal of Botany*. 95 (10): 1297-1306. https://doi.org/10.3732/ajb.0800187

[68] Westergaard, M. (1958). The Mechanism of Sex Determination in Dioecious Flowering Plants. *Advances in Genetics*. 9:217-281. https://doi.org/10.1016/S0065-2660(08)60163-7

[69] Chattopadhyay, D., and Sharma, A.K. (1991). Chromosome Studies and Nuclear DNA in Relation to Sex Difference and Plant Habit in Two Species of Cucurbitaceae. *Cytologia (Tokyo)*. 56: 409-417.

[70] Sousa, A., Fuchs, J., Renner, S.S. (2013). Molecular cytogenetics (FISH, GISH) of *Coccinia grandis*: A ca. 3 myr-old species of cucurbitaceae with the largest Y/autosome divergence in flowering plants. *Cytogenetic and Genome Research.* 139 (2): 107-118. https://doi.org/10.1159/000345370

[71] Chakravorti, A.K. (1948). Cytology of *Coccinia Indica* W. & A. with reference to the behaviour of its sex-chromosomes. *Proceedings of the Indian Academy of Sciences - Section B*. 27:74-86. https://doi.org/10.1007/BF03049901

[72] Sousa, A., Fuchs, J., Renner, S.S. (2017). Cytogenetic comparison of heteromorphic and homomorphic sex chromosomes in *Coccinia* (Cucurbitaceae) points to sex chromosome turnover. *Chromosome Research*. 25:191-200. https://doi.org/10.1007/s10577-017-9555-y

[73] Nakajima, G. (1942). Cytological Studies in Some Flowering Dioecious Plants with Special Reference to the Sex Chromosomes. *Cytologia.* 262-270.

[74] Sinha, S., Guha, A., Sinha, R.K. (2003). Karyotype and Sex Expression in *Trichosanthes dioica*,” *Cytologia (Tokyo).* 68 (4):357-361. https://doi.org/10.1508/cytologia.68.357

[75] Hu, S., Zhu, J., Ji, G., Wang, Z., Xin, J. (2019). Transcriptome sequencing and screening of genes related to sex determination of *Trichosanthes kirilowii* Maxim. *bioRxiv* [Preprint]. https://doi.org/10.1101/2019.12.17.879635

[76] Kumar, S., Singh, B.D., Sinha, D.P., Rai, M. (2008). Sex expression-associated RAPD markers in pointed gourd (*Trichosanthes dioica*). Conference paper: *Cucurbitaceae 2008, Proceedings of the IXth EUCARPIA Meeting on Genetics and Breeding. INRA, France.*

[77] Wolf, D.E., Satkoski, J.A., White, K., Rieseberg, L.H. (2001). Sex determination in the androdioecious plant *Datisca glomerata* and its dioecious sister species *D. cannabina. Genetics*. 159 (3):1243-1257.

[78] Girma, G., Natsume, S., Carluccio, A.V., Takagi, H., Matsumura, H., Uemura, A., Muranaka, S., et al. (2019). Identification of candidate flowering and sex genes in white guinea yam (*D. rotundata* Poir.) by SuperSAGE Transcriptome Profiling. *PLoS ONE*. 14 (9): e0216912 . https://doi.org/10.1371/journal.pone.0216912

[79] Tamiru, M., Natsume, S., Takagi, H., White, B., Yaegashi, H., Shimizu, M., Yoshida, K. et al. (2017). Genome sequencing of the staple food crop white guinea yam enables the development of a molecular marker for sex determination. *BMC Biology*. 15, 86. https://doi.org/10.1186/s12915-017-0419-x

[80] Bhat, B.K., Bindroo, B.B., Nagar, S. (1980). Sex chromosomes in *Dioscorea* *deltoidea* Wall. *Cytologia*. 45: 739–42.

[81] Martin, F.W. (1966). Sex ratio and sex determination in *Dioscorea*. J*ournal of Heredity*. 59 (3):95-99. https://doi.org/10.1093/oxfordjournals.jhered.a107485

[82] Terauchi, R., and Kahl, G. (2000). Rapid isolation of promoter sequences by TAIL-PCR: The 5’-flanking regions of pal and pgi genes from yams (*Dioscorea*). *Molecular and General Genetics*. 263:554-560. https://doi.org/10.1007/s004380051201

[83] Cormier, F., Lawac, F., Maledon, E., Gravillon, M.C., Nudol, E., Mournet, P., Vignes, H., Chaïr, H., Arnau, G. (2019). A reference high-density genetic map of greater yam (*Dioscorea alata* L.). *Theoretical and Applied Genetics*. 132: 1733–1744. https://doi.org/10.1007/s00122-019-03311-6

[84] Jia, H.M., Jia, H.J., Cai, Q.L., Wang, Y., Zhao, H.B., Yang, W.F., Wang, G.Y. et al. (2018). The red bayberry genome and genetic basis of sex determination. *Plant Biotechnology Journal*. 17 (12): 397-409. https://doi.org/10.1111/pbi.12985

[85] Kafkas, S., Khodaeiaminjan, M., Güney, M., Kafkas, E. (2015). Identification of sex-linked SNP markers using RAD sequencing suggests ZW/ZZ sex determination in *Pistacia vera* L. *BMC Genomics*. 16, 98. https://doi.org/10.1186/s12864-015-1326-6

[86] Sola-Campoy, P.J., Robles, F., Schwarzacher, T., Rejón, C.R., De La Herrán, R., Navajas-Pérez, R. (2015). The molecular cytogenetic characterization of pistachio (*Pistacia vera* L.) suggests the arrest of recombination in the largest heteropycnotic pair HC1. *PLoS ONE*. 10 (2): e0143861. https://doi.org/10.1371/journal.pone.0143861

[87] Turkeli, Y., and Kafkas, S. (2013). First genetic linkage map in pistachio constructed using an interspecific cross between *Pistacia vera* L. and monoecious *Pistacia atlantica* Desf. *Scientia Horticulturae*. 151:30-37. https://doi.org/ 10.1016/j.scienta.2012.11.024

[88] Khodaeiaminjan, M., Kafkas, E., Güney, M., Kafkas, S. (2017). Development and linkage mapping of novel sex-linked markers for marker-assisted cultivar breeding in pistachio (*Pistacia vera* L.). *Molecular Breeding.* 37, 98. https://doi.org/ 10.1007/s11032-017-0705-x

[89] Mathew, L.S., Spannagl, M., Al-Malki, A., Binu G., Torres, M.F., Al-Dous, E.K., Al-Azwani, E.K, et al. (2014). A first genetic map of date palm (*Phoenix dactylifera*) Reveals long-range genome structure conservation in the palms. *BMC Genomics*. 15, 285. https://doi.org/10.1186/1471-2164-15-285

[90] Torres, M.F., Mathew, L.S., Ahmed, I., Al-Azwani, I.K., Krueger, R., Rivera-Nuñez, D., Mohamoud, Y.A, Clark, A.G., Suhre, K., Malek, J.A. (2018). Genus-wide sequencing supports a two-locus model for sex-determination in *Phoenix*. *Nature Communications*, 9, 3969. https://doi.org/10.1038/s41467-018-06375-y

[91] Cherif, E., Zehdi, S., Castillo, K., Chabrillange, N., Abdoulkader, S., Pintaud, J.C., Santoni, S., Salhi-Hannachi, A., Glémin, S., Aberlenc-Bertossi, F. (2013). Male-specific DNA markers provide genetic evidence of an XY chromosome system, a recombination arrest and allow the tracing of paternal lineages in date palm. *New Phytologist*. 197 (2):409-415. https://doi.org/10.1111/nph.12069

[92] Cherif, E., S. Zehdi-Azouzi, A. Crabos, K. Castillo, N. Chabrillange, J. C. Pintaud, A. Salhi-Hannachi, S. Glémin, and F. Aberlenc-Bertossi. (2016). Evolution of sex chromosomes prior to speciation in the dioecious *Phoenix* species. *Journal of Evolutionary Biology*. 29 (8): 1513–1522. https://doi.org/10.1111/jeb.12887

[93] Al-Ani, B., Zaid, A., Shabana, H. (2010). On the status of chromosomes of the date palm (*Phoenix dactylifera* L.). *Acta Horticulturae*. 882:253-268. https://doi.org/10.17660/ActaHortic.2010.882.28

[94] Ali, H.B.M., Abubakari, A., Wiehle, M., Krutovsky, K.V. (2017). Gene-specific sex-linked genetic markers in date palm (*Phoenix dactylifera* L.). *Genetic Resources and Crop Evolution*. 65: 1-10.

[95] Telgmann-Rauber, A., Jamsari, A., Kinney, M.S., Pires, J.C., Jung, C. (2007). Genetic and physical maps around the sex-determining M-locus of the dioecious plant asparagus. *Molecular Genetics and Genomics*. 278:221-234. https://doi.org/10.1007/s00438-007-0235-z

[96] Barlow, B.A., and Wiens, D. (1976). Translocation heterozygosity and sex ratio in *Viscum fischeri*. *Heredity (Edinb).* 37 (1): 27–40.

[97] Pakull, B., Groppe, K., Meyer, M., Markussen, T., Fladung, M. (2009). Genetic linkage mapping in aspen (*Populus tremula* L. and *Populus tremuloides* Michx.). *Tree Genetics & Genomes*, 5:505-515. https://doi.org/10.1007/s11295-009-0204-2

[98] Pakull, B., Groppe, K., Mecucci, F., Gaudet, M., Sabatti, M., Fladung, M. (2011). Genetic mapping of linkage group XIX and identification of sex-linked SSR markers in a *Populus tremula* × *Populus tremuloides* cross. *Canadian Journal of Forest Research*. 41 (2):245-253. https://doi.org/10.1139/X10-206

[99] Tuskan, G.A., DiFazio, S., Faivre-Rampant, P., Gaudet, M., Harfouche, A., Jorge, V., Labbé, J.L. et al. (2012). The obscure events contributing to the evolution of an incipient sex chromosome in *Populus*: A retrospective working hypothesis. *Tree Genetics and Genomes*. 8: 559-571. https://doi.org/10.1007/s11295-012-0495-6Tuskan et al 2012

[100] Tuskan, G.A., DiFazio, S., Jansson, S., Bohlmann, J., Grigoriev, I., Hellsten, U. et al. (2006). The Genome of Black Cottonwood, *Populus trichocarpa* (Torr. & Gray). *Science*. 313 (5793):1596-1604. https://doi.org/10.1126/science.1128691

[101] Yin, T., DiFazio, S.P., Gunter, L.E., Zhang, X., Sewell, M.M., Woolbright, S.A., Allan, G.J. et al. (2008). Genome structure and emerging evidence of an incipient sex chromosome in *Populus.* *Genome Research*. 18:422-430. https://doi.org/10.1101/gr.7076308

102] Gaudet, M., Jorge, V., Paolucci, I., Beritognolo, I., Mugnozza, G.S., Sabatti, M. (2008). Genetic linkage maps of *Populus nigra* L. including AFLPs, SSRs, SNPs, and sex trait. *Tree Genetics and Genomes.* 4:25-36. https://doi.org/10.1007/s11295-007-0085-1

[103] Pucholt, P., Rönnberg-Wästljung, A.C., Berlin, S. (2015). Single locus sex determination and female heterogamety in the basket willow (*Salix viminalis* L.). *Heredity*. 114:575–583. https://doi.org/10.1038/hdy.2014.125

[104] Zhou, R., Macaya-Sanz, D., Rodgers-Melnick, E., Carlson, C.H., Gouker, F.E., Evans, L.M., Schmutz, J. et al. (2018). Characterization of a large sex determination region in *Salix purpurea L*. (Salicaceae). *Molecular Genetics and Genomics*. 293:1437-1452. https://doi.org/10.1007/s00438-018-1473-y

[105] Hou, J., Ye, N., Zhang, D., Chen, Y., Fang, L., Dai, X., Yin, T. (2015). Different autosomes evolved into sex chromosomes in the sister genera of *Salix* and *Populus*. *Scientific Reports*. 5, 9076. https://doi.org/10.1038/srep09076J.

[106] Li, W., Wu, H., Li, X., Chen, Y., Yin, T. (2020). Fine mapping of the sex locus in Salix triandra confirms a consistent sex determination mechanism in genus Salix. Horticulture Research. 7, 64. https://doi.org/10.1038/s41438-020-0289-1

[107] Sanderson, B.J., Feng, G., Hu, N., Carlson, C.H., Smart, L.B., Keefover-Ring, K., Yin, T. et al. (2020). “Sex determination through X-Y heterogamety in *Salix nigra*.” *BioRxiv* [Preprint]. Available at: https://doi.org/10.1101/2020.03.23.000919

[108] Russell, J.R.W., and Pannell, J.R. (2015). Sex determination in dioecious *Mercurialis annua* and its close diploid and polyploid relatives. *Heredity*. 114:262-271. https://doi.org/10.1038/hdy.2014.95

[109] Khadka, D.K., Nejidat, A., Tal, M., Golan-Goldhirsh, A. (2002). DNA markers for sex: Molecular evidence for gender dimorphism in dioecious *Mercurialis annua* L. *Molecular Breeding*. 9:251-257. https://doi.org/10.1023/A:1020361424758

[110] Veltsos, P., Ridout, K.E., Toups, M.A., González-Martínez, S.C., Muyle, A., Emery, O. et al*.* (2019). Early Sex-Chromosome Evolution in the Diploid Dioecious Plant *Mercurialis annua*. *Genetics*. 212 (3):815–835. https://doi.org/10.1534/genetics.119.302045

[111] Costantini, L., Battilana, J., Lamaj, F., Fanizza, G., Grando, M.S. (2008). Berry and phenology-related traits in grapevine (*Vitis vinifera* L.): From Quantitative Trait Loci to underlying genes. *BMC Plant Biology.* 8:38. https://doi.org/ 10.1186/1471-2229-8-38

[112] Marguerit, E., Boury, C., Manicki, A., Donnart, M., Butterlin, G., Némorin, A. et al*.* (2009). Genetic dissection of sex determinism, inflorescence morphology and downy mildew resistance in grapevine. *Theoretical and Applied Genetics.* 118 (7):1261-1278. https://doi.org/10.1007/s00122-009-0979-4
